# Cough-induced catastrophe: superior epigastric artery rupture causing rectus sheath hematoma

**DOI:** 10.1093/jscr/rjag679

**Published:** 2026-07-31

**Authors:** Aseel Hafaitha, Sarah Saife, Mohammad Salameh, Ihab Shraideh

**Affiliations:** Department of Medicine, Faculty of Medicine and Health Sciences, An-Najah National University, Nablus, Palestine; Department of Medicine, Faculty of Medicine and Health Sciences, An-Najah National University, Nablus, Palestine; Department of General Surgery, Rafidia Governmental Surgical Hospital, Nablus, Palestine; Department of General Surgery, Rafidia Governmental Surgical Hospital, Nablus, Palestine

**Keywords:** rectus sheath hematoma, superior epigastric artery, cough-induced hemorrhage, acute abdominal pain, computed tomography, surgical management, active extravasation, abdominal wall hematoma

## Abstract

Spontaneous rectus sheath hematoma is a rare cause of acute abdominal pain that may mimic surgical emergencies. We report a 68-year-old man with cough-induced superior epigastric artery rupture causing active hemorrhage. Computed tomography established the diagnosis, and surgical evacuation with vessel ligation achieved successful hemostasis and favorable recovery.

## Introduction

Rectus sheath hematoma (RSH) is an uncommon cause of acute abdominal pain resulting from bleeding into the rectus abdominis muscle sheath, typically due to rupture of the superior or inferior epigastric vessels or direct muscle injury. Though rare, it can present as an acute abdomen and mimic more common surgical conditions such as appendicitis or incarcerated hernia [1, 2].

RSH is most frequently associated with anticoagulant therapy, abdominal trauma, vigorous coughing, or physical strain, but spontaneous cases can occur, particularly in elderly patients or those with vascular fragility [2, 3]. The clinical spectrum ranges from mild localized pain to hypovolemic shock secondary to active hemorrhage. In some cases, ecchymosis of the abdominal wall may appear as a delayed sign [2].

The diagnosis relies heavily on computed tomography (CT) imaging, which can accurately identify the size, location, origin, extension and nature of the hematoma, and helps exclude other abdominal pathologies. Management may be conservative, interventional, or surgical depending on hemodynamic stability and imaging findings [2].

We report a case of a 68-year-old man who developed a spontaneous right rectus sheath hematoma with active extravasation from the superior epigastric artery, and was managed successfully through surgical evacuation and ligation. This case highlights the importance of early diagnosis and timely intervention, with individualized management according to clinical stability and available resources.

## Case presentation

A 68-year-old man with a history of ischemic heart disease, hyperlipidemia, and prior inguinal hernia repair presented to the emergency department with right lower quadrant pain and swelling for 3 days, worsened by walking and coughing. He had recently experienced an upper respiratory tract infection with a persistent cough for 1 week. He was not on anticoagulation but was taking aspirin, carvedilol, and atorvastatin.

On admission, the patient was afebrile and hemodynamically stable, with blood pressure of 170/80 mmHg and heart rate of 70 beats/minute. Physical examination demonstrated localized swelling and tenderness over the right lower abdominal wall without guarding, rebound tenderness, or overlying skin discoloration.

The differential diagnoses for right lower quadrant pain and swelling included incarcerated inguinal hernia, acute appendicitis, abdominal wall abscess, retroperitoneal hematoma, and rectus sheath hematoma. The absence of peritoneal signs and normal coagulation profile favored an abdominal wall etiology.

Laboratory investigations revealed hemoglobin level of 13.5 g/dL with normal coagulation parameters.

Contrast-enhanced CT of the abdomen demonstrated a right rectus sheath hematoma with active contrast extravasation originating from the superior epigastric artery, associated with surrounding soft tissue edema. An incidental infrarenal abdominal aortic aneurysm measuring 5 cm was also identified (Fig. 1).

**Figure 1:**
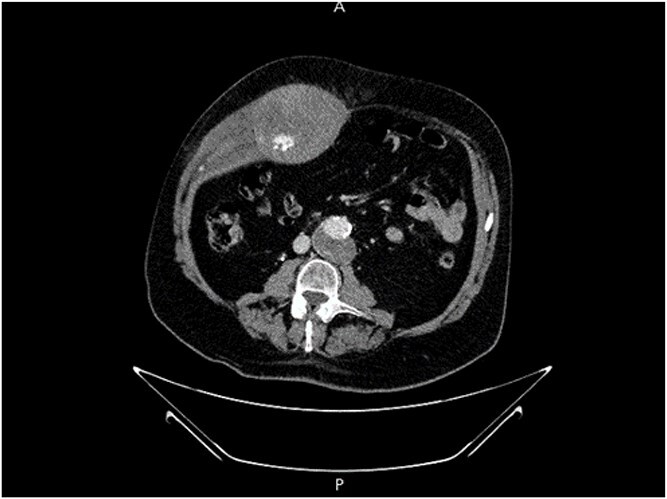
Contrast-enhanced axial CT image demonstrating a right rectus sheath hematoma with active contrast extravasation from the superior epigastric artery, consistent with ongoing bleeding.

The patient was admitted and, after prompt contrast-enhanced CT confirmed active hemorrhage, underwent urgent operative management on the day of admission. Supportive treatment included intravenous fluids, analgesia, cefuroxime, and pantoprazole.

Through a right paramedian incision, evacuation of the rectus sheath hematoma was achieved. Active bleeding from the superior epigastric artery was identified and controlled by surgical ligation. Hemostasis was secured, and a Hemovac drain was placed before layered wound closure.

Postoperatively, the patient was monitored in the intensive care unit. He remained hemodynamically stable, and drain output progressively decreased from 200 mL to 50 mL over the following 2 days. Hemoglobin stabilized at 10.9 g/dL without need for blood transfusion. Mild right-sided abdominal ecchymosis developed postoperatively without evidence of recurrent bleeding.

The drain was removed on postoperative day five, and the patient was discharged in stable condition on oral cefuroxime 500 mg twice daily for 7 days and paracetamol as needed. He was advised to undergo alternate-day wound dressing and vascular surgery follow-up for the abdominal aortic aneurysm. Two weeks after discharge, the patient developed deep vein thrombosis and received appropriate anticoagulation therapy with good clinical outcome.

## Discussion

Rectus sheath hematoma (RSH) is an uncommon but clinically significant cause of acute abdominal pain resulting from bleeding into the rectus sheath due to disruption of the epigastric vessels or direct muscular injury. [3] Although anticoagulation therapy remains the most recognized predisposing factor, spontaneous cases may occur in elderly individuals following episodes of intense coughing, sneezing, vomiting, or physical exertion. Increased intra-abdominal pressure during forceful coughing can generate sufficient shearing stress to rupture weakened epigastric vessels or tear rectus muscle fibers [4].

The clinical presentation of RSH is frequently nonspecific and may mimic several intra-abdominal emergencies including appendicitis, incarcerated hernia, diverticulitis, abdominal wall abscess, or retroperitoneal hemorrhage. Patients commonly present with localized abdominal pain, swelling, and tenderness, while ecchymosis may appear later in the disease course. Because of its variable presentation, delayed diagnosis may lead to significant morbidity, hemodynamic instability, or unnecessary exploratory surgery [5].

Radiological imaging plays a pivotal role in the diagnosis and management of rectus sheath hematoma. Ultrasonography may provide an initial bedside assessment; however, contrast-enhanced CT remains the diagnostic modality of choice because of its superior sensitivity and specificity [6]. CT imaging accurately characterizes the size, location, and extent of the hematoma while differentiating abdominal wall pathology from intra-abdominal disease. More importantly, the identification of active contrast extravasation allows detection of ongoing hemorrhage and assists clinicians in selecting the most appropriate therapeutic strategy. CT imaging also facilitates classification of hematoma severity and detection of associated complications or incidental findings, as demonstrated in this case by the concurrent abdominal aortic aneurysm [2, 4].

Management of RSH depends primarily on hemodynamic stability, hematoma size, and the presence of active bleeding. Most stable patients can be managed conservatively with analgesia, fluid resuscitation, correction of coagulopathy, and close observation. In patients with persistent bleeding, selective transcatheter arterial embolization has emerged as a minimally invasive and highly effective therapeutic option with favorable success rates [7]. Nevertheless, surgical intervention remains necessary in selected cases involving hemodynamic compromise, expanding hematoma, abdominal compartment concerns, or limited access to interventional radiology services [8].

Although transcatheter arterial embolization is an effective minimally invasive option for ongoing rectus sheath hematoma bleeding, surgery was selected in this case because CT demonstrated active extravasation, the patient proceeded to same-day exploration, and immediate surgical hemostasis was available. Surgical evacuation and ligation achieved rapid hemorrhage control and favorable postoperative recovery. This case highlights the importance of maintaining a high index of suspicion for RSH in elderly patients presenting with acute abdominal pain following severe coughing, even in the absence of anticoagulant therapy or trauma. Early radiologic diagnosis and individualized treatment are essential to optimize outcomes and prevent potentially life-threatening complications.

## Conclusion

Rectus sheath hematoma is an uncommon but important cause of acute abdominal pain that may mimic intra-abdominal emergencies. Although typically associated with anticoagulation or trauma, it may also occur spontaneously after forceful coughing. This case emphasizes the importance of early diagnosis, prompt imaging, and individualized management, ranging from conservative care to embolization or surgical ligation when appropriate. Timely recognition and intervention are essential for favorable outcomes.

## Data Availability

All data supporting this case report are contained within the manuscript. Additional details are available from the corresponding author upon reasonable request.
